# Anoikis classification of lung squamous cell carcinoma reveals correlation with clinical prognosis and immune characteristics

**DOI:** 10.1080/07853890.2025.2514944

**Published:** 2025-06-14

**Authors:** Yixin Zhai, Cheng Li, Xiang He, Wenqi Wu, Donghui Xing, Kaiping Luo, Zhigang Zhao

**Affiliations:** a Department of Hematology, Tianjin Medical University Cancer Institute and Hospital, National Clinical Research Center for Cancer, Key Laboratory of Cancer Prevention and Therapy, Tianjin’s Clinical Research Center for Cancer, Tianjin, China; b Department of Senior Ward, Tianjin Medical University Cancer Institute and Hospital, National Clinical Research Center for Cancer, Key Laboratory of Cancer Prevention and Therapy, Tianjin’s Clinical Research Center for Cancer, Tianjin, China; c Department of Medical Oncology, Tianjin First Central Hospital, School of Medicine, Nankai University, Tianjin, China

**Keywords:** Anoikis1, clinical prognosis5, gene expression omnibus(GEO)3, immune characteristics 6, lung squamous cell carcinoma (LUSC)2, The Cancer Genome Atlas (TCGA)4

## Abstract

**Background:**

Anoikis is a new mode of cell death that has been shown to correlate significantly with tumors. However, the clinical prognostic significance of anoikis in lung squamous cell carcinoma (LUSC) remains poorly studied.

**Methods:**

The differentially expressed ARGs and candidate genes were selected by the differential analysis to construct a predictive model. Independent prognostic gene was determined by Cox and LASSO analysis and we used the HCC95 and NCI H520 cell line to verify the gene function. We used the data from TCGA, GEO, GeneCards, and Harmonizome databases to analyze the immune microenvironment, functional enrichment, and drug sensitivity analysis.

**Results:**

We identified 717 differentially expressed and selected 3 ARGs (FADD, SNAI1, and BAG4) to construct a predictive model. We found that SNAI1 is an independent prognostic gene and confirmed that knocking out the SNAI1 inhibited the HCC95**/**NCI H520 cell proliferation. We used single-sample gene-set enrichment analysis (ssGSEA) to evaluate the immune infiltration based on the 3 ARG expression levels. We constructed a risk score and provided a visual representation of the prophetic implications of the ARGs-based signature through a nomogram. We found 15 susceptible drugs in the high-risk group and 15 sensitive drugs in the low-risk group by the drug sensitivity analysis.

**Conclusion:**

We used ARGs to construct a prognosis model for LUSC that can accurately predict the prognosis of LUSC patients. ARGs, especially SNAI1, play an essential role in developing LUSC. These findings could provide individualized treatment plans and new research ideas for LUSC patients.

## 1. Introduction

Lung cancer poses a significant challenge to global public health, with global cancer data indicating that more than 2 million people are diagnosed with lung cancer each year, including approximately 236,740 new cases reported in the United States in 2022.[1]. Recently, there has been a gradual upward trend in the number of reported lung cancer cases in China [2]. Among lung cancer classifications, approximately 80% to 85% of non-small cell lung cancer (NSCLC) and according to histopathology features, lung squamous cell carcinoma (LUSC) has the second highest incidence [1].

Relevant studies have shown that the occurrence of cancer metastasis is associated with increased cancer mortality [3]. The process of tumor metastasis involves developing circulating tumor cells (CTCs), forming secondary tumors, and metastatic establishment [4]. However, when cell adhesion is reduced, tumor-migrating cells face difficulties in survival [5]. Therefore, the survival and death of tumor cells are closely related to the extracellular matrix. The extracellular matrix (ECM) is crucial in cell differentiation, proliferation, and movement [6]. The ECM comprises various proteins, including collagens, glycoproteins, proteoglycans, elastin, laminins, and fibronectins. Alterations in the structure of ECM-associated proteins are associated with the onset and progression of various pathologies[7]. Enhanced NSCLC cell metastasis is associated with elevated levels of certain ECM elements [8]. Meanwhile, the extracellular matrix (ECM) is an essential element of the tumor microenvironment. Cells thrive in typical tissue environments, but when confronted with unfavorable conditions, they often undergo programmed cell death.

Anoikis is a type of programmed cell death triggered when a cell loses its attachment to the appropriate extracellular matrix, ultimately leading to a break in the integrin linkage [9]. This process is essential for maintaining tissue homeostasis and preventing the survival of detached cells, which could otherwise lead to pathological conditions. The extracellular matrix influences tumorigenesis and development in various cancer types, playing the role of a cellular regulator [10]. Alterations in the extracellular matrix in cancer, stromal, and immune cells can promote tumor cells’ proliferation, survival, and spread [11–13]. Research indicates that the expression of ECM-related proteins, particularly tenascin-c, has been more extensively investigated in lung adenocarcinoma than in squamous cell carcinoma [13]. The studies about anoikis have been reported in other tumors and diseases[14–17], but its role in LUSC is unclear.

In the context of cancer, anoikis resistance is a critical factor that enables tumor cells to survive and proliferate in distant sites, facilitating metastasis [18,19]. Tumor cells that evade anoikis can survive in the bloodstream or lymphatic system, where they are exposed to various immune cells. Anoikis resistance not only aids in the survival of these circulating tumor cells but also enhances their ability to evade immune surveillance, thereby contributing to immune escape. This resistance is often associated with changes in the tumor microenvironment, such as epithelial–mesenchymal transition (EMT), which endows tumor cells with greater motility and anoikis resistance.

Moreover, anoikis resistance is linked to treatment resistance in cancer. Tumor cells that can resist anoikis are more likely to survive chemotherapy or targeted therapies, leading to poor treatment outcomes. For example, studies have shown that specific genes associated with anoikis resistance, such as ETV7 in pancreatic cancer, can serve as prognostic markers and potential therapeutic targets [18]. Understanding the mechanisms of anoikis resistance can provide valuable insights into developing new strategies to combat cancer metastasis and improve treatment efficacy.

Consequently, this research aimed to establish an innovative predictive framework for LUSC through a bioinformatics strategy focusing on genes associated with anoikis and the immune response.

## 2. Materials and methods

### 2.1. Data acquisition

We used publicly available datasets in this study. 504 clinical samples from TCGA, which include 51 normal samples and 502 tumor samples, with one sample having been excluded. 43 LUSC samples were sourced from the GSE50081 dataset, 238 samples from GSE30219, GSE73403 and GSE37745. 49976 transcriptome profiles data retrieved from GDC TCGA. The patients diagnosed with LUSC were characterized by various clinical factors such as age, sex, tumor grade, TMN staging, duration of survival, and their status regarding survival. For this analysis, this data is secondary data and does not contain any data which can identify individual. Therefore, ethics statement has been exempted by the Ethical Review Committee of Tianjin Medical University Cancer Institute and Hospital.

### 2.2. Analysis of gene differential expression and establishment of prognostic network

We employed the BioManager and limma packages to analyze the differentially expressed genes (DEGs) in normal and tumor samples in anoikis–LUSC TCGA data. The screening criteria of DEGs is |log2(fold change)| >1 and adjusted FDR < 0.05. We selected the top 50 up-regulated and 50 down-regulated genes for our study. The heatmap and volcano plot was performed with ‘limma’ and ‘pheatmap’ packages of R software. We used the survival package, limma package, survminer package, reshape2 package, and RColorBrewer package in R. Adjusted *p* < 0.05 was considered statistically significant. Anoikis–TCGA and GEO clinical data were obtained from uniCOX analysis expressed by forest, and the data expression through prognostic networks.

### 2.3. Consensus clustering analysis for anoikis-related genes

A total of 717 genes associated with anoikis were identified and categorized through the official GeneCards website (https://genecards.org/) and the Harmonizome platform (https://maayanlab.cloud/Harmonizome/). The genomic data for TCGA LUSC were sourced from the official UCSC Xena website (https://xena.ucsc.edu/). The investigation analyzed copy number variations (CNV), genetic mapping, and the somatic mutation rates for the identified anoikis-related factors. The mutation frequency derived from CNV was computed utilizing a Perl script algorithm, and the data was visually represented using the ‘RCircos’ tool.

We used the software package ‘Consensus Cluster Plus’ R to perform a consistency analysis and clustered and scored the data according to the expression profiles of anoikis-related genes. We used the k-means algorithm to classify TCGA–LUSC and GSE50081 data into several clusters. According to the consensus matrix, consistent cluster score, and cumulative distribution function (CDF) curve, we determined the maximum subtype number *k* (*k* = 2) and thoroughly assessed the optimal number of clusters.

### 2.4. Functional enrichment analysis and immune evaluation of different molecular subtypes

We conducted the analysis of genomic variations to inquire the functional relationships between different groups. The data of ‘c2.cp.kegg.symbols.gmt’ and ‘c5.go.symbol s.gmt’ from MSigDB could be used to functional enrichment analysis. Genomic pathways could be identified by using the R ‘GSVA’ package. Adjusted *p* < 0.05 was considered as statistically significant. We used R ‘GSEABase’ and ‘GSVA’ packages to achieve the immune assessment. We used the R ‘limma’ package to analyze immune-related genes between clusters and used the R ‘ggpubr’ and ‘heatmap’ packages to draw the boxplot and heat map analysis results. We used R ‘clusterProfiler’ and ‘enrichplot’ packages for GO/KEGG enrichment analysis and results visualization respectively.

### 2.5. Establishment and verification of anoikis-related prognostic model

We established a model to assess the association between the LUSC prognosis and the anoikis. Firstly, we applied univariate analysis to recognize survival-related genes in differentially expressed genes (DEGs) based on the cluster. Secondly, an anoikis-associated prognostic risk model was been developed by using Lasso–Cox analysis. Next, we divided all the LUSC samples into risk train set (*n* = 269) and risk test set (*n* = 269) randomly, and calculated the anoikis risk score by the model equation, which according to the three essential gene expression profiles (FADD, BAG4, SNAI1). Model Formula: Risk score =** **FADD* 0.156653387181982-BAG4* 0.194369656312506 + SNAI1* 0.174563712695362. We used Kaplan–Meier survival analysis to evaluate the effect of risk score in clinical prognosis between high-risk and low-risk groups, which were defined according to the standard classification rules. The accuracy of the 1-, 3-, and 5-year predictions for the train, test, and all groups was verified by receiver-operator characteristic (ROC) curves. Principal component analysis (PCA) was performed based on the expression of the anoikis-related characteristic genes. The distribution of the groups was explored using t-distributed stochastic neighbor (t-SNE) analysis using the ‘Rtsne’ ‘R package’. Additionally, the dataset GSE30219, GSE73403 and GSE37745 serves to validate the anoikis model.

### 2.6. Construction and verification of nomogram

We processed the data by constructing independent prognostic analyses using the R ‘survival’ and ‘survminer’ packages. Adjusted *p* < 0.05 was considered as statistically significant. Forest plots plotted the results of the independent prognostic analyses. The entire risk expression file and typing results were expressed through the heatmap package by drawing the risk heatmap. We constructed a nomogram based on the multiple clinical characteristics and risk scores of LUSC patients, which can show the corresponding 1-, 3- and 5-year survival probabilities based on the different risk scores. We used the ‘rms’ R package to make this nomogram, and used the ‘ggpubr’ R package to draw the Box plot. Observing the relationship between sample typing and risk scores through the Sankey diagram to express the accuracy of patient survival in 1-, 3- and 5-year periods by drawing calibration curves. We used the ‘ggDCA’ R package to construct decision curves in 1-, 3- and 5-year periods.

### 2.7. Molecular subtypes and the tumor microenvironment

We used the CIBERSORT algorithm to assess the characteristics of immune cell infiltration within the tumor microenvironment (TME) (https://cibersort.stanford.edu/about.php), which categorizes different immune cell types. These findings from the CIBERSORT analysis formed the basis for the subsequent evaluations. We examined the immune cell profiles within the anoikis risk classification. Utilizing the ‘limma’ R package, we assessed the heatmap illustrating the relationships between risk scores and immune checkpoint genes. The data regarding immune cell infiltration and risk scores were processed using the R package ‘limma’. The disparities between high-risk and low-risk groups were illustrated through bar charts created with the ‘ggpubr’ package, correlation visualizations utilizing the ‘ggpubr’ library for ordered gene expression data.

Moreover, we assessed the variations in TME scores and the expression of immune-related genes across the two risk categories. Subsequently, we estimated the tumor-infiltrating immune cell (TIC) abundance profile and immune-related biological functions using a single-sample (ssGSEA) algorithm. Adjusted *p* < 0.05 was considered as statistically significant.

### 2.8. Analysis of tumor mutation score and drug sensitivity

Thee violin diagrams were used to visually demonstrate the differences between stromalScore, ImmuneScore, and ESTIMATEScore risk groups. We used expression data and sensitivity data of targeted drugs from Genetics of Drug Sensitivity in Cancer (GDSC)(https://www.cancerrxgene.org/). To obtain score files of the sensitivity of the train and test groups to the different therapeutic drugs, we firstly analyzed the differential expression data of the anoikis gene in TCGA and GEO for drug sensitivity by using R software including ‘limma’, ‘oncoPredict’, and ‘parallel’ packages. And then, the obtained drug susceptibility score files and sample risk files were processed using the ‘ggplot’ and ‘ggpur’ software packages, and the drug susceptibility results for the high-risk and low-risk groups were plotted draw box plots. Multifactorial results will be available, including FADD, SNAI1, and BAG4.

### 2.9. Identification of independent prognostic gene

Independent prognostic genes were identified through the analysis of univariate and multivariate Cox regression, and forest plot were used to display the variate (*p* value, hazard ratios (HR), and 95% confidence intervals (CI). The Kaplan–Meier survival analysis can enable to show prognostic difference between high and low levels of independent prognostic gene expression. *p* < 0.05 was considered statistically significant.

### 2.10. Knocking out SNAI1

The LUSC cell lines HCC95/NCI H520 and 293 T were purchased from the American Type Culture Collection (ATCC). The lentiviral particles were generated using 293 T cell lines. The sequence of sgRNA that targets SNAI1 is as follows: SNAI1-sg2: ctctgaggccaaggatctcc; The alternative sgRNA designed for SNAI1 is noted as SNAI1-sg6: gaagtagaggagaaggacga. Following the knockout of SNAI1, we tested the genome indel, mRNA relative expression, proliferation, and apoptosis in cells.

### 2.11. Statistical analysis

For this study, we use *p* values and *p* < 0.05 was considered to be statistically significant (*p < 0.05; **p < 0.01; ***p < 0.001; ****p < 0.0001). We used a univariate Cox proportional risk regression model to calculate risk ratios for univariate analyses and a multivariate Cox regression model to determine independent prognostic factors. Significance of differences in risk scores at different stages assessed by Wilcox test. All analyses were conducted with R version 4.3.0 in the present study. Related R packages including ‘survival’, ‘BioManager’, ‘limma’, ‘heatmap’, ‘survminer’, ‘reshape2’, ‘RColorBrewer’, ‘RCIRCOS’, ‘consensus cluster plus’, ‘Rtsne’, ‘umap’, ‘ggplot’, ‘ggpubr’, ‘pheatmap’, ‘GSEABase’, ‘GSVA’, ‘GSEBase’, ‘clusterProfiler’, ‘enrichplot’, ‘caret’, ‘glmnet’, ‘timeROC’, ‘ggplot2’, ‘ggalluvial’, ‘dplyr’, ‘regplot’, ‘rms’, ‘ggDCA’, ‘corrplot’, ‘vioplot’, ‘tidyverse’ and ‘ggExtra’ were downloaded from the Lanzhou University Open-Source Society.

## 3. Results

### 3.1. Differential expression of anoikis-related genes and altered transcriptional activity in LUSC/identification of differentially expressed anoikis-related genes based on LUSC subtype analysis

**Figure 1.**
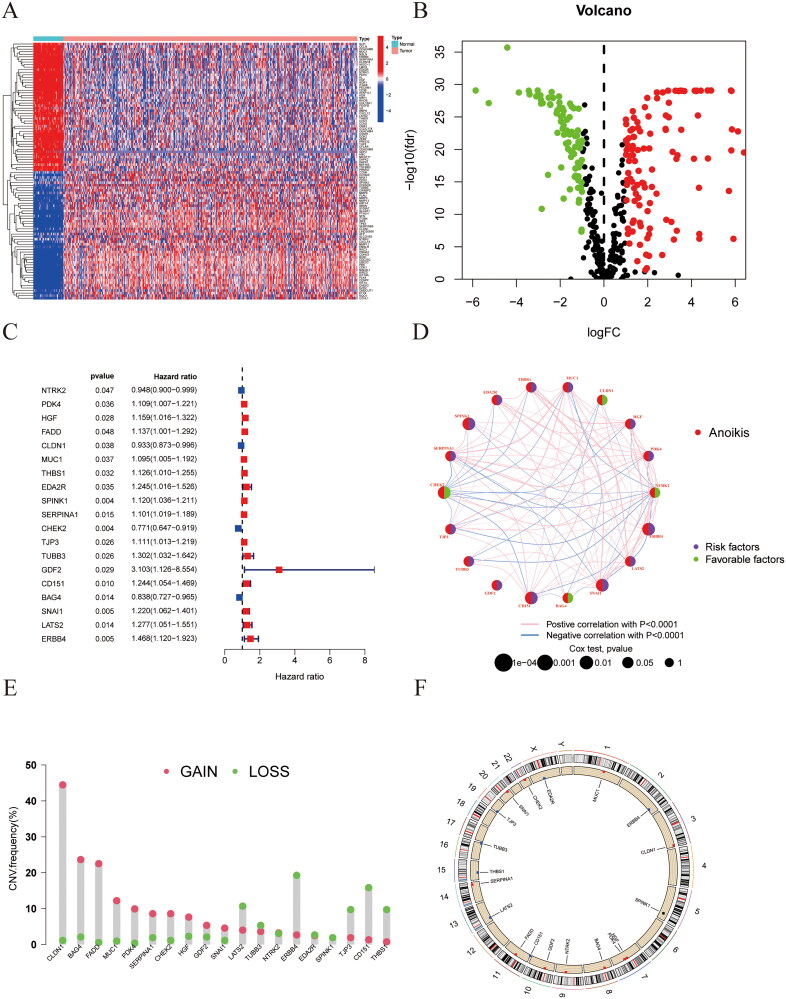
Screening of prognostic-related genes and mutation frequency analysis. (A) Gene expression heat map of differentially expressed anoikis-related genes in Normal and Tumor subtypes. (B) Volcano plot showing the differentially expressed anoikis related genes in tumors versus normal tissue samples. (C) The forest plot shows the top 19 ARGs (*p* < 0.01) via the univariate Cox regression analysis. (D) Network diagram showed the correlations between the top ARGs. (E) Copy number variations (CNVs) of 19 ARGs. (F) Chromosome region and alteration of ARGs.

The investigation into the significance of genes associated with anoikis in the context of LUSC was conducted. The study provided insights into genes’ differential expression and subsequent transcriptional activity. A comprehensive analysis revealed 407 genes associated with anoikis, identified from the differences between the normal and diseased cohorts in the TCGA dataset. then we selected 197 differentially expressed genes (DEGs) and used the thermal map shows that 50 ARGs were up-regulated and 50 ARGs were down-regulated genes between anoikis -normal and anoikis-tumor subtypes based on the screening criteria of |logFC(fold change)|>1, FDR < 0.05 (Figure 1A). The volcanic map shows the distribution of these ARGs (Figure 1B; Supplementary Table 1). A total of 19 out of 197 differentially expressed genes (DEGs) were identified as related to overall survival (OS) in the univariate Cox regression analysis, as illustrated in the forest plot (Figure 1C; Supplementary Table 2). The study of anoikis-related genes by network graph displays the association of risk factors based on *p* values (Figure 1D). Regarding copy number variations, a higher amplification frequency was observed in 10 genes than in deletions. Specifically, CLDN1, BAG4, FADD, MUC1, PDK4, SERPINA1, CHEK2, HGF, GDF2, and SNAI1 showed extensive CNV amplification. in contrast, several genes like LATS1、ERBB4、SPINK1、TJP3、CD151、and THBS1 were found to display deletions in their CNV (Figure 1E). The graphical representation of changes in CNV across 19 anoikis-related genes is illustrated on the chromosomes (Figure 1F).

### 3.2. Identification of a classification pattern of LUSC based on the phenotype of anoikis

**Figure 2.**
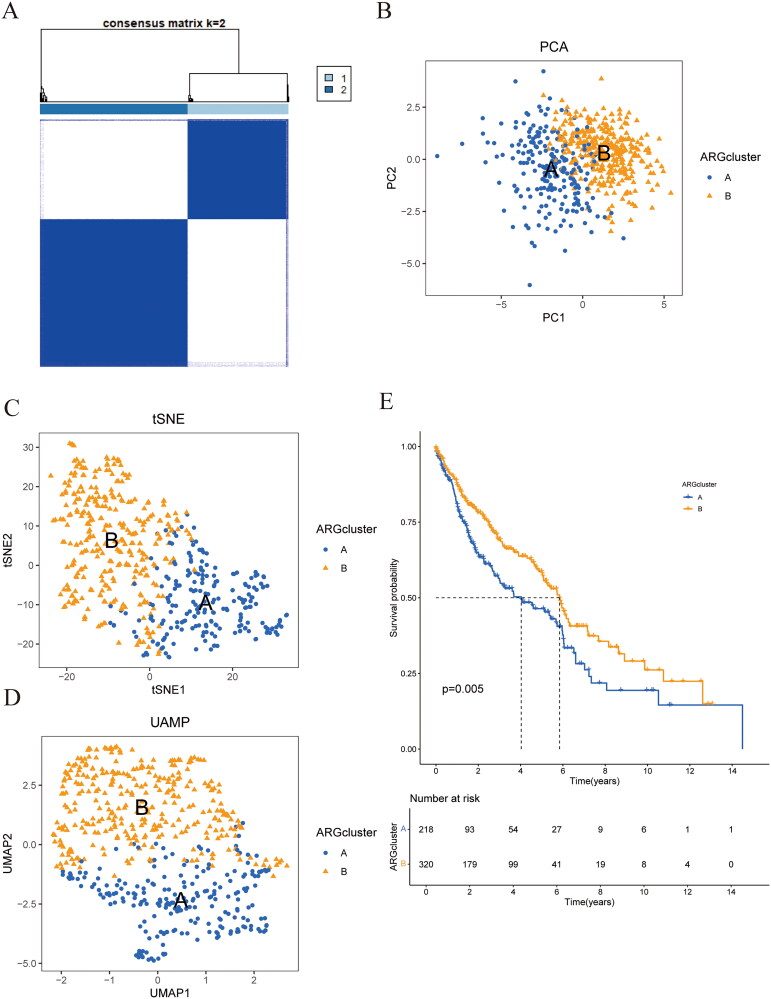
Analysis of consensus cluster. (A) The consensus matrixes *k* = 2 was obtained by applying consensus clustering. (B) PCA analysis showed differences between the two subtypes. (C) tSNE analysis showed differences between the two subtypes. (D) UAMP analysis showed differences between the two subtypes. (E) The Kaplan–Meier survival curves showed differences between the two subtypes

From the TCGA–LUSC and GEO-GSE50081 datasets, a total of 19 factors related to anoikis were identified. A consensus clustering algorithm was employed to categorize patients with LUSC based on the expression data of the 19 identified genes. To explore the expression patterns of genes associated with anoikis in LUSC, a total of 544 samples derived from 501 cases in TCGA–LUSC along with 43 samples from GEO-GSE50081 were analyzed using the consensus clusterplus approach, focusing on the expression data of 19 anoikis-related genes. According to our findings, *k* = 2 was the best grouping variable for dividing the dataset into Cluster A and Cluster B (Figure 2A). Cluster analysis revealed two distinct groups of anoikis-associated genes, designated as Group A with 220 samples and Group B consisting of 324 samples. Dimensionality reduction techniques such as PCA, tSNE, and UAMP analysis indicated a pronounced clustering of samples from types A and B (Figure 2B–D). According to the survival analysis, the survival results of the two clusters were significantly different (*p* = 0.005) (Figure 2E).

### 3.3. The immune infiltration and biological functional analysis of different anoikis subtypes

**Figure 3.**
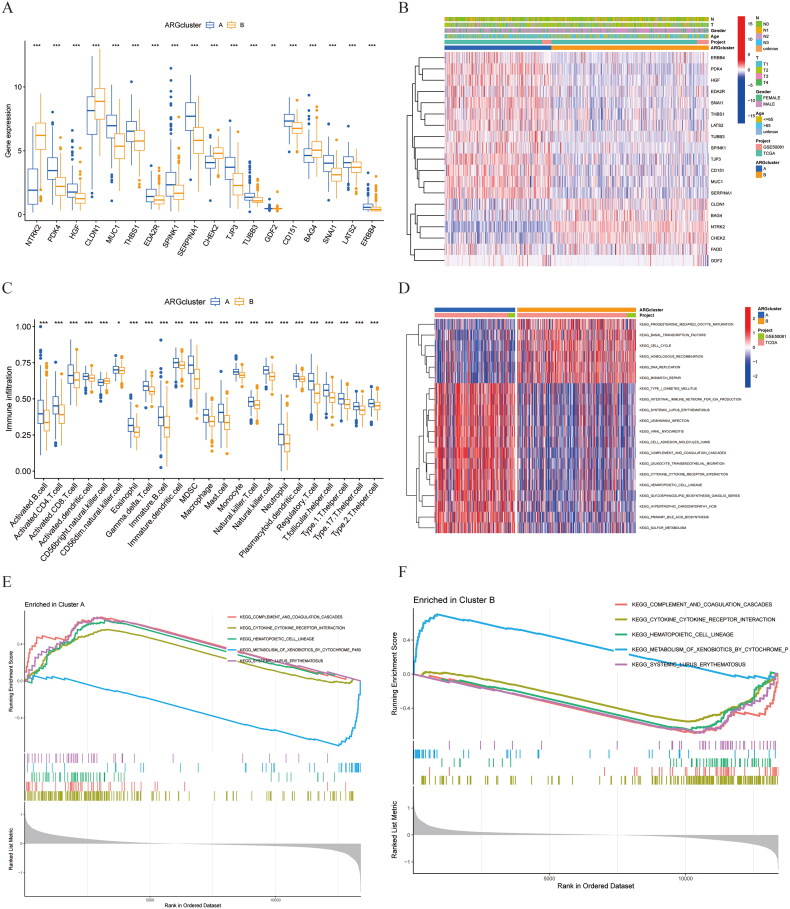
The immune infiltration and biological functional analysis of different anoikis subtypes. (A) ARGs expression in two subtype clusters. (B) Heatmap of ARGs expression and corresponding clinicopathological features of two subtypes. (C) Immune infiltration patterns in two subtype clusters. (D–F) GSVA and GSEA analysis focused on the differential enrichment of KEGG pathways between clusters B and A.

Various assessments indicated that these four genes exhibited elevated expression levels in cluster B, as supported by the heatmap analysis (Figure 3A,B). Additionally, two distinct subtypes exhibited a notable accumulation of immune cells (Figure 3C). A GSVA analysis was performed to compare the biological activities between clusters A and B (Figure 3D). In cluster B, significant enrichment was observed for pathways such as KEGG_PROGESTERONE_MEDIATED_OOCYTE_MATURATION. KEGG_BASAL_TRANS­CRIPTION_FACTORS, KEGG_CELL_CYCLE, KEGG_HOMO­LOGOUS_RECOMBINATION. KEGG_DNA_REPLICATION. and KEGG_MISMATCH_REPAIR, while the other pathways showed strong enrichment in cluster A (Figure 3D). In addition, an analysis of the pathways involved in clusters A and B was conducted using Gene Set Enrichment Analysis (GSEA), revealing that the genes significantly expressed in cluster A were predominantly associated with KEGG_COMPLEMENT_AND_COAGULATION_CASCADES, KEGG_CYTOKINE_CYTOKINE_RECEPTOR_INTERACTION. KEGG_HEMATOPOIETIC_CELL_LINEAGE, and KEGG_SYS­TEMIC_LUPUS_ERYTHEMATOSUS (Figure 3E). The analysis indicated that KEGG_METABOLISM_OF_XENOBIOTICS_BY_CYTOCHROME_P450 was notably prevalent in Cluster B (Figure 3F).

### 3.4. Construction and validation of the anoikis prognostic model

**Figure 4.**
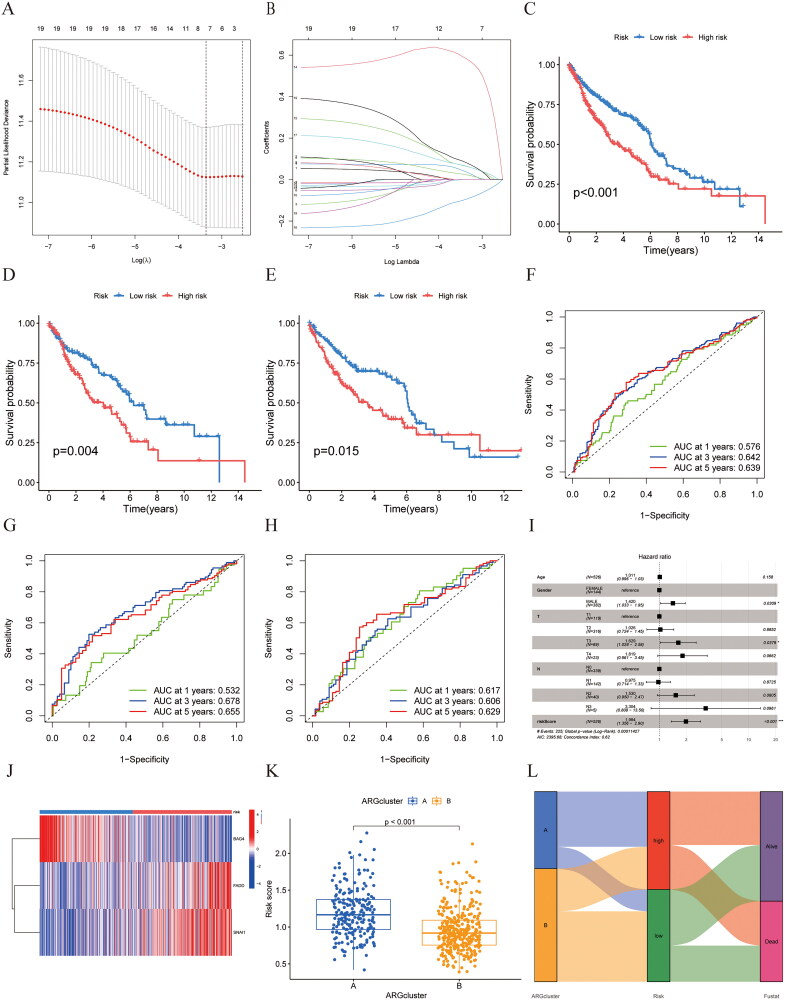
Construction of a prognostic model. (A, B) LASSO regression analysis shows the minimum lambda and optimal coefficients of the prognostic ARGs. (C–E) The K–M curves showed different prognosis in the different risk groups. (C) All group (D) Train group (E) Test group. (F–H) The time-dependent ROC curves for OS at 1-, 3-, and 5-years. (F) All group (G) Train group (H) Test group. (I) Multivariate Cox regression analysis of the risk score and other clinicopathological factors. (J) Heatmap diagram shows the expression of the 3 prognostic ARGs. (K) Risk score in two clusters. (L) Alluvial diagram of subtype and living status.

The categorization of anoikis holds significant relevance for the clinical outcomes associated with LUSC. A prognostic model was developed as an initial step to gain deeper insights into the predictive traits of anoikis. A total of 717 genes associated with anoikis were discovered from the clusters, and through univariate Cox analysis, 19 genes linked to prognosis were identified. Secondly, Lasso–Cox regression analysis was conducted on the 19 prognostic genes identified (Figure 4A,B), and three significant genes were discovered (FADD, SNAI1, and BAG4 (Supplementary Table 3). The risk score prognostic model were constructed according to the three anoikis-related genes. Risk score** **=** **FADD* 0.156653387181982-BAG4* 0.194369656312506 + SNAI1* 0.174563712695362. Then, The LUSC samples were classified into high-risk and low-risk categories according to their risk scores, revealing that the prognosis for those in the high anoikis risk category was less favorable than that of the low anoikis risk category (Figure 4C, *p*< 0.001). This result was confirmed by Kaplan–Meier curves of the train and test groups (Figure 4D–E, *p* = 0.004, *p* = 0.015). We also confirmed the high accuracy of the anoikis risk score in predicting prognosis after 1, 3, and 5 years. The area under the curve (AUC) for the total sample was 0.576 over 3 and 5 years. 0.642 and 0.639 for 1, 3, and 5 years (Figure 4F). The AUC in the training cohort was determined to be 0.532, 0.678, and 0.655 for 1, 3, and 5 years (Figure 4G). The AUC values recorded in the evaluation set were 0.617, 0.606, and 0.629 for 1, 3, and 5 years (Figure 4H). Additionally, we used the external cohort (the dataset GSE30219, GSE73403, and GSE37745) to validate the anoikis model further. The results also showed the prognostic ability of the anoikis model and were statistically significant (Supplementary Figure 1). Considering the age in the analysis, Gender, T N M classification, the risk score was associated with OS (HR = 1.984, 95% confidence interval 1.356–2.90, *p* < 0.001) (Figure 4I). The risk heatmap showed that BAG4 was a low-risk gene, and FADD and SNAI1 were identified as genes associated with high risk (Figure 4J). The analysis of differences reveals distinct characteristics between the patients in clusters A and B, highlighting the relationship between patient categories and their outcomes (Figure 4K). Ultimately, the findings highlight the promising ability of the anoikis risk score as a prognostic indicator. The alluvial diagram revealed that the patients in the low-risk category and most of those who survived predominantly belong to subtype B. In contrast, those exhibiting elevated risk scores are classified under subtype A, consistent with earlier findings (Figure 4L).

### 3.5. The nomogram plot construction of the ARG model

**Figure 5.**
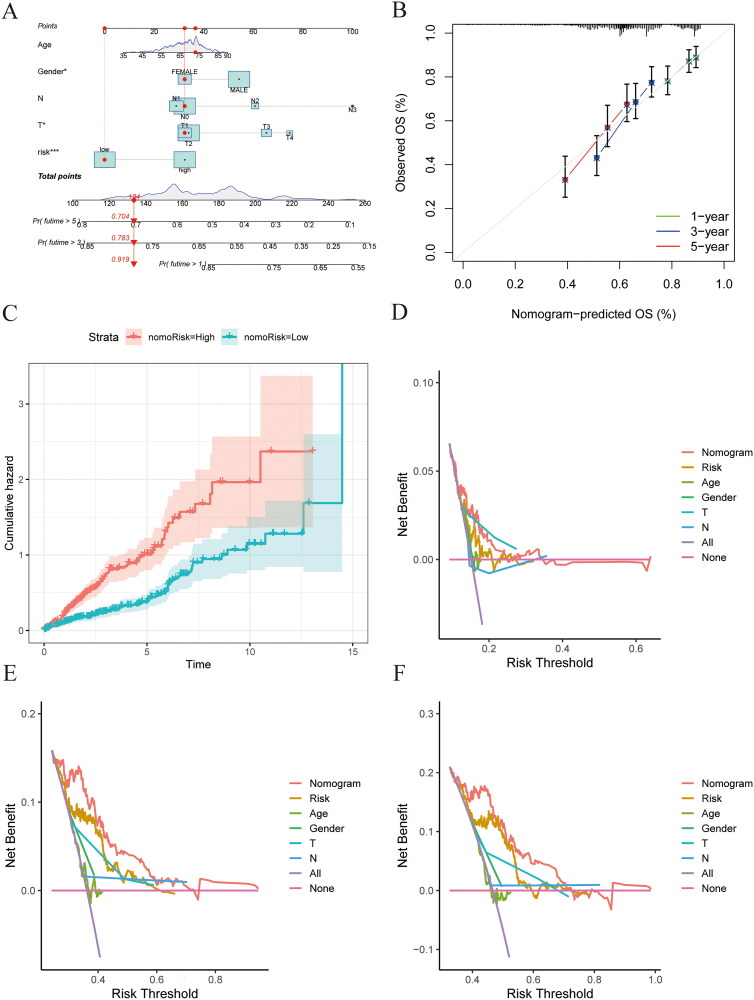
The nomogram plot construction of the ARGs model. (A) Nomogram plot predicting 1,3, and 5-year overall survival in patients with LUSC. (B) Calibration plot for the validation of nomogram. (C) Cumulative hazard curve represented the probability of survival over time progression. (D–F) DCA curves of the nomogram for 1-, 3- and 5-year OS in LUSC patients.

We integrated the risk scores and various clinical and pathological variables to enhance the prediction accuracy for the LUSC prognosis. Utilizing the developed nomogram, the overall scores derived from the clinical data can yield a survival rate percentage for patients at 1, 3, and 5 years (Figure 5A). The survival rates for patients at the 1, 3, and 5-year marks were determined to be 0.919, 0.783, and 0.704. The calibration graph indicates that the predicted outcomes for the 1-year, 3-year, and 5-year overall survival rates based on the nomogram closely aligned with the ideal scenario for the entire cohort (Figure 5B). The survival curves indicate that individuals classified in the high-risk category faced a heightened risk compared to their low-risk counterparts (Figure 5C). The DCA analysis revealed that the developed nomogram outperformed other clinicopathological variables in forecasting survival outcomes for patients with LUSC (Figure 5D–F).

### 3.6. Construction of risk score and tumor microenvironment analysis

**Figure 6.**
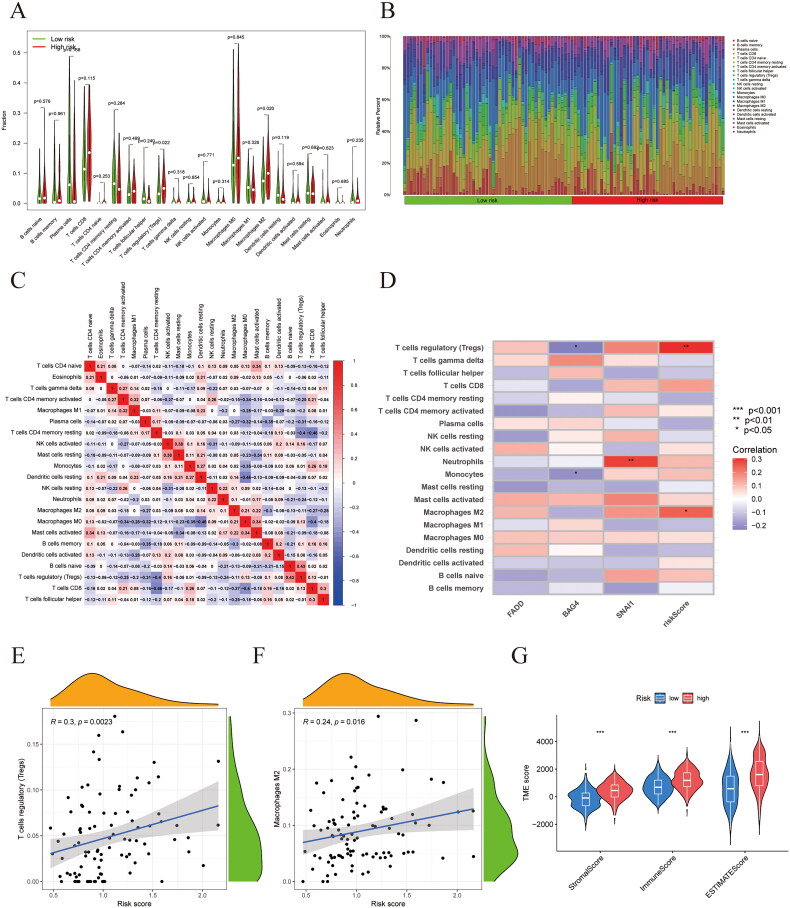
Immune cell infiltration and tumor microenvironment analysis. (A) Immune cell component between the high-risk group and low-risk group. (B) The relative proportion of infiltrating immune cells with different risk scores. (C) The correlation between immune cells. (D) The correlation of immune cells, Anoikis genes, and risk score. (E, F) The correlation analysis between risk score and the proportion of immune cells. (G) Estimate score of the expression profile in the high-risk group and low-risk group.

To investigate the association between the infiltration of innate immune cells (ICs) and anoikis-related genes, we employed the CIBERSORT algorithm to assess the distribution of these immune cells (Figure 6A,B). The analysis presented in the heatmap illustrates how ICs are associated with the anoikis genes (Figure 6C,D). A notable positive association was observed between the risk score and regulatory T cells (Tregs) and M2 macrophages (Figure 6E,F). Analysis of the differential scores within the tumor microenvironment (TME) revealed notable disparities between the groups classified as high-risk and low-risk regarding StromalScore, ImmuneScore, and ESTIMATEScore. Specifically, the TME score was markedly elevated in the high-risk cohort compared to the low-risk cohort (Figure 6G).

### 3.7. Drug sensitivity analysis

**Figure 7.**
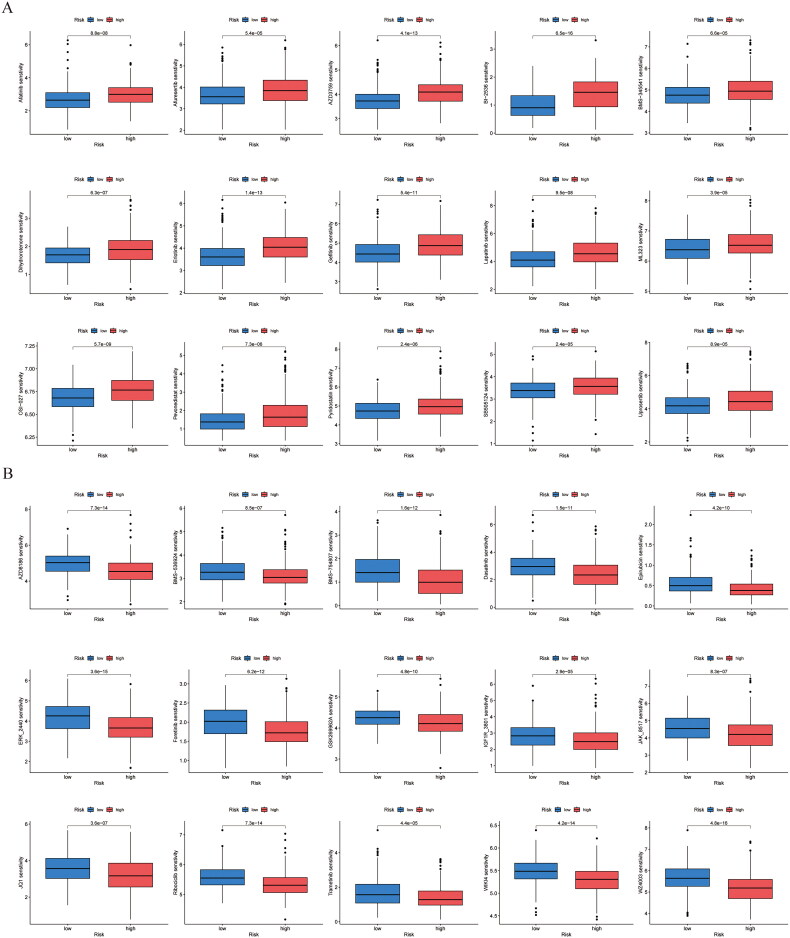
Drug sensitivity analysis. IC50 values were calculated for patients in the high- and low-risk groups to assess the sensitivity of chemotherapeutic agents. (A) Drugs that are highly sensitive in low-risk groups. (B) Drugs that are highly sensitive in high-risk groups.

The use of pharmacological treatments is vital in managing tumors. Subsequently, an analysis was carried out regarding the sensitivity of drugs and the prognostic biomarkers in patients with LUSC, revealing notable differences in 30 drugs between the high-risk and low-risk categories. The IC50 values for a selection of 15 pharmacological agents (Afatinib, Afuresertib, AZD3759, BI-2536, BMS-345541, Dihydrorotenone, Erlotinib, Gefitinib, Lapatinib, ML323, OSI-027, Pevonedistat, Pyridostatin, SB505124, Uprosertib), were found to be elevated in individuals identified as high-risk, suggesting that the vulnerability to these medications in the high-risk category was less compared to that observed in the low-risk category (Figure 7A), with the IC50 values for the 15 studied medications (AZD8186, BMS-536924, BMS-754807, Dasatinib, Epirubicin, ERK-2440, Foretinib, GSK269962A, IGF1R-3801, JAK-8517, JQ1, Ribociclib, Trametinib, WIKI4, WZ4003) were markedly reduced in individuals classified as high-risk, indicating that these medications have a strong efficacy in individuals categorized as high risk (Figure 7B).

### 3.8. Screening of independent prognostic genes and their preliminary validation

**Figure 8.**
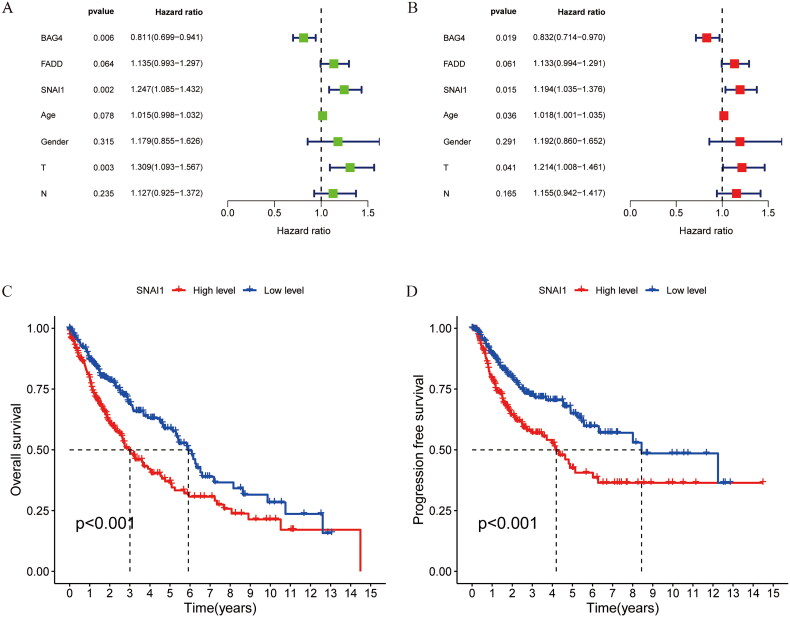
Screening for independent prognostic genes. (A,B) Hazard ratio and p-value of the constituents involved in univariate and multivariate Cox regression considering clinical parameters and three prognostic ARGs in LUSC. (C,D) Comparison of OS and PFS in SNAI1 high expression group and low expression group.

Subsequently, univariate and multivariate Cox regression analysis was conducted on the expressions of the three prognostic genes and clinical factors, focusing on *p* values, risk coefficients (HR), and confidence intervals (CI). The analysis indicated that BAG4, SNAI1, and the T category of TNM classification may serve as independent prognostic indicators for LUSC (Figure 8A,B). The study of the Kaplan–Meier curves indicated that individuals with high levels of SNAI1 expression experienced a poorer prognosis than those with lower levels, with the findings reaching statistical significance (Figure 8C,D).

### 3.9. Validation of SNAI1 prognostic function in LUSC

**Figure 9.**
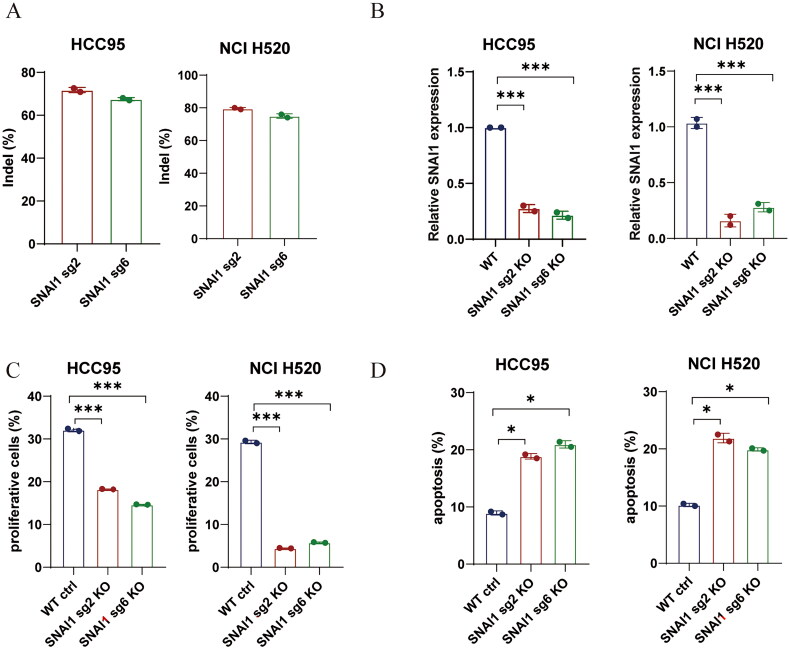
Validation of SNAI1 prognostic function in LUSC. (A) Indel activity of Cas9 at two sites of the SANI1 locus measured by sanger sequencing. (B) Flow cytometric analysis of EdU staining after SNAI1 knockout. (C) Bar plots of cell proliferation.

The SNAI1 gene was disrupted in the HCC95 and NCI H520 cell line using CRISPR technology with sgRNA to assess the prognostic value of SNAI1 in lung squamous cell carcinoma (LUSC). We tested the genome indel efficiency by sanger sequencing (Figure 9A), and tested the SNAI1 mRNA relative expression by qPCR (Figure 9B). The proliferation was inhibited prominently and apoptosis was significantly increased in cells of knocking out the SNAI1 (Figure 9C,D). In conclusion, these findings indicate that the prognostic gene SNAI1 is crucial for the proliferation of LUSC.

## 4. Discussion

Anoikis is essential for ensuring that cells maintain appropriate placement within tissues, thereby preventing them from attaching to incorrect sites, which can lead to abnormal growth[18]. Anoikis disrupts the connection between cells and the surrounding extracellular matrix. In the context of lung cancer, the biological functions of cells and the extracellular matrix play a critical role in the process of anoikis. The main factor contributing to this phenomenon is triggering signals that lead to cell death. Conversely, stimulation of proliferative signaling can serve to prevent anoikis [5].

Nonetheless, existing studies are needed to explore further the significance of anoikis-related genes, particularly for cancer aggressiveness, degree of immune cell infiltration, resistance to LUSC therapies, and the potential of anoikis-related genes as predictors. The investigation sought to clarify the significant influence of anoikis on the onset and advancement of LUSC and how it shapes the immune environment within the LUSC microenvironment through an extensive examination of both transcriptomic and proteomic data. We constructed a prediction model of anoikis-related genes, in which three anoikis-related genes were associated with LUSC, which were BAG4, FADD, and SNAI1. These three anoikis genes are related to LUSC and are found in the extracellular matrix. They are also expressed in colorectal, ovarian, and cervical cancers[19]. The model can categorize LUSC patients into high-risk and low-risk groups, which can be used for subsequent studies.

The correlation analysis of the three genes is as follows. The protein known as Bcl2-associated athanogene 4 (BAG4) is a component of the human BAG protein family, often referred to as an inhibitors of death domains. BAG4 has a variety of cellular functions, including the process of programmed cell death, cell growth, reorganization of cell structure, cell mobility, breakdown of cellular components, and the ability to penetrate other tissues. Research has indicated that BAG4 is associated with invasive characteristics of a variety of human tumor types, including pancreatic, breast, acute lymphoblastic leukemia, gastric, and colon cancers [20–22]. However, the implications in clinical practice and the potential molecular mechanisms of BAG4 concerning LUSC remain to be thoroughly examined. The results of this study suggest that BAG4 may be an essential gene associated with anoikis in LUSC, which is valuable for prognostic prediction. The SNAI1 gene is a transcription factor that affects the expression of E-cadherin, mainly by promoting the shift to a mesenchymal phenotype, which in turn enhances the invasion and migration of cancer cells through the epithelial–mesenchymal transition (EMT) process [23]. It has been shown that inhibition of the SNAI1 pathway by GATA6-AS1 is associated with tumor proliferation and lung metastasis in pancreatic ductal adenocarcinoma (PDAC). As demonstrated by *in vivo* studies, GATA6-AS1 may decrease the stability of SNAI1 mRNA, thereby hindering the proliferation, invasion, migration, and metastasis of PDAC cells [24].

P450 was primarily engaged in cluster B. The complement system is a critical component of the immune response and the mechanism by which tumors evade immune surveillance. An increased presence of the complement-inhibitory proteins CD55 and CD59 has been observed in non-small cell lung cancer (NSCLC) among various other malignancies, which has been associated with signaling from cytokines [25]. In human lung cancers, the levels of CD55 and CD59 were found to be adversely related to the infiltration of immune cells that combat tumors and indicated a worse prognosis. Reducing the expression of CD55 and CD59, which EGFR heightens, can trigger the complement system and enhance lung cancer sensitivity to checkpoint inhibitors.

Utilizing combined therapies of antibodies targeting both CD55/CD59 and programmed death 1 (PD-1) results in a collaborative effect that inhibits tumor growth [26]. Additionally, the expression levels of HOXA3 genes are interconnected with pathways linked to focal adhesion and ECM–receptor interactions. The focal adhesion pathway is essential for activating focal adhesion kinase (FAK), which influences various cellular metabolic functions, including cellular movement, the signaling processes induced by growth factors, cell survival progression through the cell cycle, and cell movement, which has a significant connection to the advancement of malignant tumors. Some studies indicate that using FAK as a potential target for targeted therapy in NSCLC could be beneficial. The signaling pathway that interacts with ECM receptors is also implicated in tumor spread and infiltration processes. It also facilitates the growth and movement of cancer cells.

Nevertheless, further exploration of the two pathways linked to LUSC remains necessary[27]. Numerous studies have indicated the significance of SHOX2 across various cancer types, including lung cancer, Matt. The detection of SHOX2 methylation can potentially identify early-stage lung cancer patients [28]. Nevertheless, the significance of SHOX2 in patients with lung squamous cell carcinoma and the underlying molecular mechanisms remain unclear. Therefore, examining GSVA and GSEA can lay the groundwork for advancing immunotherapy and chemotherapy strategies for LUSC.

An analysis of drug sensitivity demonstrated that 30 drugs exhibit sensitivity, 15 cases were allergic to drugs, while 15 drugs showed resistance within the high-risk group. AZD8186, a specific inhibitor targeting PI3Kβ/δ, demonstrated effectiveness against tumors in preclinical models lacking PTEN [29]. The synergistic application of AZD8186 alongside Selumetinib (a MEK/ERK pathway inhibitor) resulted in diminished cell proliferation and heightened apoptosis in docetaxel-resistant metastatic castration-resistant prostate cancer cells that express PTEN. In *in vivo* assessments, mice bearing docetaxel-resistant xenografts treated with AZD8186 and selumetinib showed a decline in tumor growth while exhibiting no additional toxicity [30]. BMS-536924 is a mighty small molecule blocker of IGF-IR, demonstrating efficacy against various cancer models, including Ewing’s sarcoma. Rhabdomyosarcoma and neuroblastoma exhibit increased sensitivity to BMS-536924. These particular subtypes could serve as potential targeted demographics for the IGF-IR inhibitor [31]. BMS-754807, functioning as antagonists for both IGF1R and IR, it was established that these inhibitors could effectively reduce the viability of diffuse midline glioma tumor cells at specific concentrations [32]. The challenge of using BRAF and MEK inhibitors in treating patients with BRAF-mutant melanoma arises from the issue of acquired resistance. Therefore, studies focusing on trametinib and dabrafenib-resistant melanoma (TDR) cell lines were conducted, and it observed that BMS-754807 hindered cellular growth and led to a decrease in intracellular p-Akt levels within TDR cells [33]. Nevertheless, the effectiveness of these agents in treating LUSC is still significantly under-researched. The current investigation aims to address this gap. The response of these medications to LUSC was evaluated, which opens up additional therapeutic avenues for LUSC treatment.

The study revealed that three specific genes, namely BAG4, FADD, and SNAI1, serve as prognostic indicators for LUSC. The selection of these genes involved the application of the LASSO method in both univariate and multivariate Cox regression analyses, leading to the development of a prognostic risk model. The established risk model demonstrates a high level of accuracy in forecasting the outcomes for patients with LUSC. SNAI1 serves as a crucial prognostic marker for LUSC, as experimental findings in LUSC demonstrated that the depletion of SNAI1 led to a marked reduction in cellular growth, emphasizing its significant contribution to LUSC. SNAI1 serves as a crucial prognostic marker for LUSC, as experimental findings in LUSC demonstrated that the depletion of SNAI1 led to a marked reduction in cellular growth and cell apoptosis increased significantly, emphasizing its significant contribution to LUSC.

The current study also has several limitations. The reliance solely on publicly available databases may introduce biases into the results. Additionally, the relatively small sample size could limit the generalizability of the findings. Future research could address these limitations by incorporating larger, multicenter datasets or conducting prospective clinical studies to validate the findings, thereby mitigating these limitations and enhancing the robustness and applicability of the results.

Ultimately, we developed a model defining the characteristics of ARGs, which offers a means to forecast the outcomes for patients with LUSC. The insights gained from this work may pave the way for innovative research directions and present new treatment avenues for healthcare providers dealing with LUSC cases.

## 5. Conclusion

This investigation employed a robust multi-omics approach to examine how changes in anoikis influence the intricate regulatory frameworks associated with LUSC. This analysis highlighted the role of genes related to anoikis in affecting the prognosis of LUSC. In this study, we identified 19 anoikis-related genes associated with survival in LUSC patients, and three anoikis genes were finally selected for modeling to predict the prognosis of LUSC cases effectively. Additionally, initial immune infiltration and functional enrichment analyses uncovered associations between various subtypes, their immune microenvironments, and distinct functional roles. The study of drug responses identified 15 drugs that showed high sensitivity in the high-risk cohort, alongside another set of 15 drugs that were highly sensitive in the low-risk cohort. The presence of SNAI1 serves as a significant prognostic indicator for LUSC, and functional assays demonstrated that the downregulation of SNAI1 markedly reduced cell growth, underscoring its critical involvement in LUSC. Ultimately, the study underscored the crucial influence of anoikis on LUSC progression, offering avenues for research and clinical exploration into its mechanisms and prospective therapeutic interventions.

## Supplementary Material

Supplemental Material

## Data Availability

The datasets generated and analyzed during the current study are available in the Cancer Genome Atlas (TCGA) database (https://portal.gdc.cancer.gov/). The datasets generated during and analyzed during the current study are available from the corresponding author upon reasonable request.
