# Supplementary material for: Anoikis classification of lung squamous cell carcinoma reveals correlation with clinical prognosis and immune characteristics
Source: Ann Med. 2025 Jun 14;57(1):2514944. doi: 10.1080/07853890.2025.2514944 (PMC12168404; doi:10.1080/07853890.2025.2514944)
Supplement: Supplemental Material [file IANN_A_2514944_SM8995.zip › suppl_data/legend.docx]

Supplementary Table 1 diffARGexp

Supplementary Table 2 forest plot

Supplementary Table 3 Multifactorial Cox regression analysis of prognosis-related ARGs.

Supplementary Figure 1: The external validation cohort of the prognostic model.

(A) The K-M curves showed different prognosis in the different risk groups.

(B) The time-dependent ROC curves for OS at 1-, 3-, and 5-years.

.
