# Supplementary figures and images for: Anoikis classification of lung squamous cell carcinoma reveals correlation with clinical prognosis and immune characteristics
Source: Ann Med. 2025 Jun 14;57(1):2514944. doi: 10.1080/07853890.2025.2514944 (PMC12168404; doi:10.1080/07853890.2025.2514944)

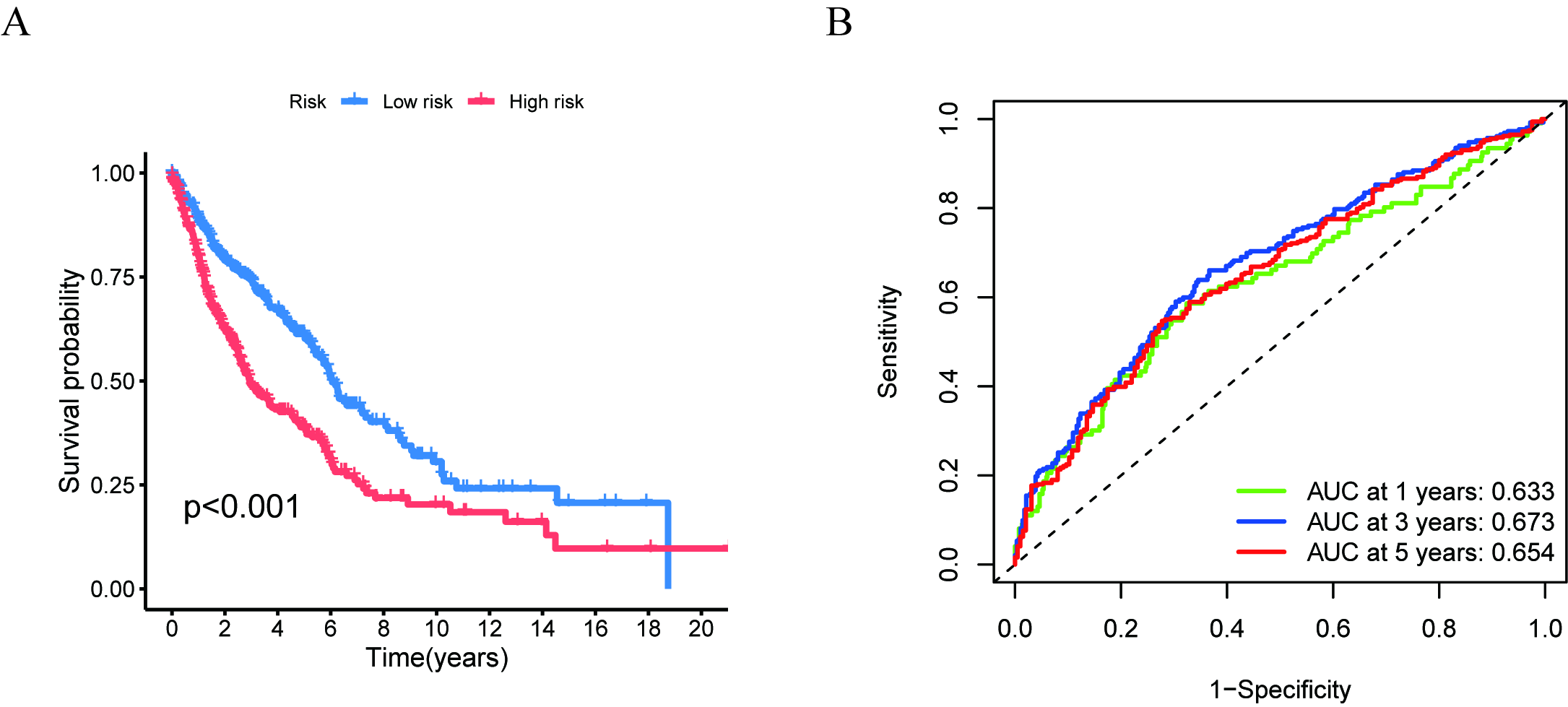

Supplement: Supplemental Material [file IANN_A_2514944_SM8995.zip › suppl_data/Supplementary Figure1.tif]
